# Emotional intelligence, emotional labor, and job satisfaction among physicians in Greece

**DOI:** 10.1186/1472-6963-12-463

**Published:** 2012-12-17

**Authors:** Aristea Psilopanagioti, Fotios Anagnostopoulos, Efstratia Mourtou, Dimitris Niakas

**Affiliations:** 1 Internal Medicine Department, University Hospital of Patras, Rion, Greece; 2 Hellenic Open University, Faculty of Social Sciences, Bouboulinas 57-59, Patras, Greece; 3 Department of Psychology, Panteion University, 136 Syngrou Avenue, Athens, Greece

**Keywords:** Emotional intelligence, Emotional labor, Surface acting, Job satisfaction, Greece

## Abstract

**Background:**

There is increasing evidence that psychological constructs, such as emotional intelligence and emotional labor, play an important role in various organizational outcomes in service sector. Recently, in the “emotionally charged” healthcare field, emotional intelligence and emotional labor have both emerged as research tools, rather than just as theoretical concepts, influencing various organizational parameters including job satisfaction. The present study aimed at investigating the relationships, direct and/or indirect, between emotional intelligence, the surface acting component of emotional labor, and job satisfaction in medical staff working in tertiary healthcare.

**Methods:**

Data were collected from 130 physicians in Greece, who completed a series of self-report questionnaires including: a) the Wong Law Emotional Intelligence Scale, which assessed the four dimensions of emotional intelligence, i.e. Self-Emotion Appraisal, Others’ Emotion Appraisal, Use of Emotion, and Regulation of Emotion, b) the General Index of Job Satisfaction, and c) the Dutch Questionnaire on Emotional Labor (surface acting component).

**Results:**

Emotional intelligence (Use of Emotion dimension) was significantly and positively correlated with job satisfaction (*r=.42, p<.001*), whereas a significant negative correlation between surface acting and job satisfaction was observed *(r=−.39, p<.001)*. Furthermore, Self-Emotion Appraisal was negatively correlated with surface acting *(r=−.20, p<.01)*. Self-Emotion Appraisal was found to influence job satisfaction both directly and indirectly through surface acting, while this indirect effect was moderated by gender. Apart from its mediating role, surface acting was also a moderator of the emotional intelligence-job satisfaction relationship. Hierarchical multiple regression analysis revealed that surface acting could predict job satisfaction over and above emotional intelligence dimensions.

**Conclusions:**

The results of the present study may contribute to the better understanding of emotion-related parameters that affect the work process with a view to increasing the quality of service in the health sector.

## Background

There is increasing evidence to suggest a positive relationship between physician job satisfaction and patient satisfaction as well as health outcomes, i.e. continuity of care, lower no-show rates, and enhanced adherence to treatment [1-4]. Interestingly, the “affective revolution”, taking place in organizational context the last decades, has pointed out the importance of psychological constructs implicated in the process of job satisfaction (JS), such as Emotional Intelligence (EI) and Emotional Labor (EL) [5].

Although emotions constitute a common characteristic of human beings, each individual differs widely in “the ability to monitor one’s own and others’ feelings and emotions, to discriminate among them and to use this information to guide one’s thinking and actions” (i.e. EI) [6], p. 189]. Mayer and Salovey [7] conceptualized four facets in EI: appraisal of emotion in self, recognition of emotion in others, regulation of emotion, and use of emotion to promote performance. Theoretical approaches were followed by the design of measures to assess the construct of EI. On the basis of the measurement method used to operationalize them, EI constructs can be categorized into trait EI (emotion-related self-perceived abilities and behavioral tendencies measured through self-report tests) and ability EI (emotion-related cognitive abilities that should be assessed via maximum-performance tests) [8]. Most scientific research in various fields is conducted within the framework of trait EI [9]. Irrespective of the theoretical framework used for empirical data interpretation, self-report measures remain important as well as widely used tools in different scientific fields [9,10].

EI has emerged as an interesting topic in social and organizational psychology [11] and appears to play a critical role in key organizational outcomes, such as job performance and JS, especially when the focus is on human interaction [12-15]. Importantly, in the health care setting, physicians who are more competent in recognizing emotions, concerns and needs of patients are more successful in treating them [15,16]. Therefore, the interpersonal communication between the patient and the physician plays a major role in patient outcomes, and emotionally intelligent physicians consist of a valuable resource for hospitals. In the organizational psychology literature, much attention has been drawn to the positive association between EI and JS (the latter being defined as “a pleasurable or positive emotional state resulting from the appraisal of one's job or job experiences”) [17], p.1304]. As supported by a wide range of studies in varied work environments, employees with higher EI are more satisfied with their job [13,14,18-21]. Furthermore, research findings from the limited number of empirical studies examining the moderating role of gender in the EI-JS relationship are controversial. According to Petrides and Furnham [22] and Salim et al. [23], gender does not moderate the path from EI to JS, whereas Afolabi et al. [24] argue that EI and gender may interact to influence JS.

H1a: EI is positively related to JS

H1b: Gender may moderate the positive effect of EI on JS

During interpersonal transactions, service employees are frequently involved in the process of emotional labor, i.e. amplifying, suppressing or faking emotions to comply with organizationally desired rules and complex role demands [25-28]. Two types of EL acting mechanisms have been proposed: surface acting (SA) and deep acting [29,30]. In SA, employees alter the outward appearance of an emotion, i.e. put on a fake smile towards an annoying customer, thereby masking true feelings. In deep acting, employees modify internal feelings in order to comply with the appropriate organizational display rules, by making an effort to understand and sympathize with other people [30]. EL is associated with emotional exhaustion and job burnout [29,31], higher levels of work stress and psychological distress, and job dissatisfaction [29,32,33]. Typically, research indicates a negative correlation between EL and JS for employees who engage in the process of SA [25,29,34-36]. According to a meta-analysis underlying the importance of each type of EL, SA, as an “arduous” process entailing both emotional suppression and production of the appropriate emotion [37], is negatively related to JS, whereas deep acting does not display any significant relationship with JS [38]. Interestingly, Johnson and Spector [39] indicated gender as a significant moderator in the relationship between SA and JS, with females being more likely to experience job dissatisfaction when engaged in SA. Furthermore, to the authors’ best knowledge, it has not been clarified whether SA is uniquely associated with JS beyond other influential factors including EI.

H2a: SA is negatively related to JS

H2b: Gender moderates the negative effect of SA on JS

H2c: SA may predict JS above and beyond EI

EI is a critical factor in performing EL; attributes of EI, such as perception and regulation of emotion, may modify employee’s EL behaviors [40,41]. Although emotionally intelligent people are assumed to be more adaptive in regulating emotions according to situational demands [7], results of studies exploring the association between EI and EL have been contradictory. Austin et al. [42] and Mikolajczak et al. [40] showed a negative correlation between EI and surface-acting EL, whereas Brotheridge [43] demonstrated no significant correlation between EI levels and SA, assuming that sample characteristics might have weakened the strength of the relation between EI and EL.

H3: EI is negatively associated with the SA component of EL.

Apart from a direct relationship between EI and JS, research has also established the mediating role of different variables, such as positive and negative affect, as well as personal accomplishment, in the EI-JS relationship [13,41]. According to Wong and Law [44], a significant positive correlation exists between EI and JS; yet, the relationship is not moderated by EL. Lee and Ok [41] recently suggested that SA played no mediating role in the EI-JS relationship in hotel employees. Given the limited evidence, any possible mediating or moderating role of SA in the EI-JS relationship remains to be elucidated.

H4a: SA may mediate the EI-JS relationship

H4b: SA may moderate the EI-JS relationship

Research has primarily focused on direct associations among organizational psychology variables, while empirical studies integrating the constructs of EI, JS and EL in healthcare occupational setting and particularly among physicians are very limited; thus no safe conclusions can be drawn. The present study investigated the possible direct and/or indirect links between EI, SA component of EL, and JS as well as any possible moderating role of SA and demographic variables in medical staff working in tertiary health care in Greece. Apart from the primary research hypotheses, reliability and validity of constructs were also tested.

## Methods

### Sample

The sample included 130 physicians, 80 males and 50 females, working at the University Hospital of Patras. Participants were administered a series of self-report questionnaires. The study protocol was approved by the Hospital Research Ethics Committee (119/25.11.10) and participation was voluntary. Response rate was 86.7%.

### Measures

#### Emotional intelligence

EI was measured using the Wong & Law Emotional Intelligence Scale (WLEIS) [44] which comprised 16 items. This scale, consistent with Mayer and Salovey’s definition of EI [7], assessed the four dimensions of EI: (a) Self-Emotion Appraisal (SEA), defined as the person’s perceived ability to understand his/her own emotions (e.g. “I really understand what I feel”), (b) Others’ Emotion Appraisal (OEA), defined as an individual’s perceived ability to understand other peoples’ emotions (e.g. “I have a good understanding of the emotions of people around me”), (c) Use of Emotion (UOE), defined as the perceived tendency to motivate self to enhance performance (e.g. “I always encourage myself to try my best”), and (d) Regulation of Emotion (ROE), defined as individuals’ perceived ability to regulate their own emotion (e.g. “I have good control of my own emotions”). Each item of the WLEIS was answered on a 7-point Likert-type scale (1=totally disagree to 7= totally agree). Cronbach’s alpha coefficients for the four EI dimensions were obtained in this study as follows: .844 for SEA, .800 for OEA, .804 for UOE, and .802 for ROE.

### Job satisfaction

JS was assessed using the Brayfield & Rothe General Index of Job Satisfaction (e.g. “I am satisfied with my job for the time being”) [45]. The scale comprises 18 items (Cronbach’s alpha equal to .947), answered on a 5-point Likert scale (1=totally disagree to 5= totally agree).

### Emotional labor

Surface Acting of EL was assessed using the first five items of the Dutch Questionnaire on Emotional Labor D-QEL (e.g. I put on a “mask” in order to express the right emotions for my job) [46] (Cronbach’s alpha equal to .846). All items were answered on a 5-point Likert scale ranging from 1 (totally disagree) to 5 (totally agree).

### Statistical procedures

Since our study was cross-sectional, common method bias could provide an alternative explanation for the correlations observed between measures of different constructs (e.g., among SEA, OEA, UOE, ROE, EL, and JS). Common method bias refers to variance that is attributable to the measurement methods used (e.g., common forms of data collection such as self-report questionnaires, common scale types, similar scale anchors and response format, item content overlap, common rater effects), rather than to the constructs the measures represent [47]. Common method bias is a main source of systematic measurement error. In order to rule out the possibility of common assessment method bias, two statistical techniques were applied: Harman’s single-factor test and the single-method-factor technique [47]. In the former case, all observed variables/ items from all the constructs in the study were included into an exploratory factor analysis (using unrotated principal component analysis as well as principal component analysis with varimax rotation) to determine whether the majority of the variance in the variables could be accounted for by one general factor. If a single factor emerges or one general factor accounts for most of the covariance among the variables, then a significant common method variance effect is present. In the latter case, items were allowed to load on their theoretical constructs, as well as on a latent common methods variance factor, applying confirmatory factor analysis (CFA). The significance of the structural parameters was then examined both with and without the latent common methods variance factor in the model [47]. One of the advantages of this latter approach is that it enables the researcher to account for measurement error in variables.

In order to assess convergent validity of the constructs, the average variance extracted (AVE) was calculated. AVE equals the sum of all squared standardized factor loadings (obtained from CFA) divided by the number of items. Fornell and Larcker [48] suggested adequately convergent valid measures of each latent construct should contain less than 50% error variance (i.e., AVE should be 0.5 or above, signifying that, on average, the variance due to measurement error is less than the variance captured by an underlying factor). Construct reliability (CR) was also used as an indicator of convergent validity. CR was computed from the squared sum of factor loadings for each construct and the sum of the error variance terms for a construct. CR estimates equal to 0.7 or higher suggest good reliability [48]. Moreover, AVE was used to evaluate discriminant validity of the constructs [49]. Discriminant validity of a target factor was established if the squared interconstruct correlations associated with that factor were less than the AVE estimates corresponding to the target factor and all the other factors, suggesting that the target factor had more internal (extracted) variance than variance shared between the factors. Discriminant validity of a target factor was further established when correlations with other constructs were (in absolute value) below 0.7, providing evidence of measure distinctness.

Regarding the relationship between EI and JS, a mediated model was developed and tested, in which EI was posited to positively influence JS both directly and indirectly through EL. Subsequently, a moderated mediation model was tested [50], in which any significant mediated effects were assumed to be moderated by gender. Furthermore, a moderated model was developed and tested, in which a significant interaction between EI and EL in predicting JS was assumed. Prior to analyses, all continuous measures were mean-centered by subtracting the variable’s mean from each case’s value on that variable, whereas gender was coded 0 for men and 1 for women. Age, years at work, position, and days of duty were included as covariates in regression equations examining mediator effects.

To assess whether mediation was present in the general theoretical model, the significance of the indirect effects of EI on JS through EL was tested using the bias-corrected bootstrap confidence intervals (CIs). Bootstrapping is a non-parametric resampling method that can be extended to designs involving indirect effects. In the case of simple mediation, indirect effects equal the product of two unstandardized regression coefficients, one representing the effect of EI (independent variable) on EL (mediator), and the other representing the effect of EL (mediator) on JS (dependent variable) controlling for EL [51]. However, using the product of regression coefficients for making inferences about indirect effects, involves implicit assumption that the sampling distribution of the indirect effect is normal. There are reasons to suspect that this assumption does not hold when mediation is present [52]. Thus, bootstrapping has been recommended. To bootstrap the sampling distribution of the indirect effects, the regression coefficients are repeatedly estimated *k* times with bootstrap samples, each of which contains *n* cases randomly sampled with replacement from the original sample (that is a given case can be selected multiple times), where *n* is the size of the original sample. This process yields *k* estimates of the indirect effects of the independent variable (EI) on the dependent variable (JS). These *k* values of the indirect effects are then sorted from low to high, thus enabling the specification of the lower and upper bounds of the desired CI [52]. MacKinnon, Lockwood, and Williams [53] conducted simulation studies to examine the accuracy of various tests on mediation effects, and advocated the bias-corrected approach as the best way to test indirect paths in mediation analysis, when normality assumptions appear to be violated. The bias-corrected bootstrap was conducted in SPSS using PROCESS computational tool [54], generating 10,000 bootstrap samples and 95% bias-corrected CIs for indirect effects. Since the percentile bootstrap CIs can be asymmetrical because they are based on an empirical estimation of the sampling distribution of the indirect effect, a correction is applied to the percentile values of the sorted distribution of bootstrap estimates used for determining the bounds of the interval. Hence the term “bias-corrected” is derived from this adjustment made to the percentile values so that the CIs are equidistant from the point estimate.

Hierarchical multiple linear regression analysis was also performed to examine the relationships between a set of independent variables (i.e., SA) and a dependent variable (i.e., JS), controlling for the effects of demographic (e.g., age, gender), work-related variables (e.g., days of duty), and other psychological variables (i.e., EI components) on the dependent variable. Screening of the raw data before they were analyzed included detection of univariate and multivariate outliers (based on the studentized residuals and the Mahalanobis distance). A search was also conducted, focused on residuals, to check for violations of the assumptions of normality, equality of variance (homoscedasticity), and linearity. Independence of error terms and sequential correlation of adjacent errors was tested through the Durbin-Watson statistic. This test statistic can vary between 0 and 4, has an acceptable range of values from 1.50 to 2.50, with a value of 2 meaning that the residuals are uncorrelated. The presence of multicollinearity was detected through inspection of the tolerance (<.10) associated with each independent variable [55].

## Results

### Sample profile

Of all participants, 61.5% (*n*=80) were males. Approximately 71% of physicians were between 30 and 39 years old, and 13.1% were between 40 and 49. Slightly over half of respondents (50.8%) were married. All of them were six year university graduates and 27.7% were PhD holders. Physicians were employed in internal medicine (48.4%), laboratory (30.8%), and surgical (20.8%) sector; 69% of them were occupied as residents. Mean value of days of duty, including weekends, in a month was 6 (ranging from 0 to 9; SD= 2).

### Common method variance

**Figure 1:**
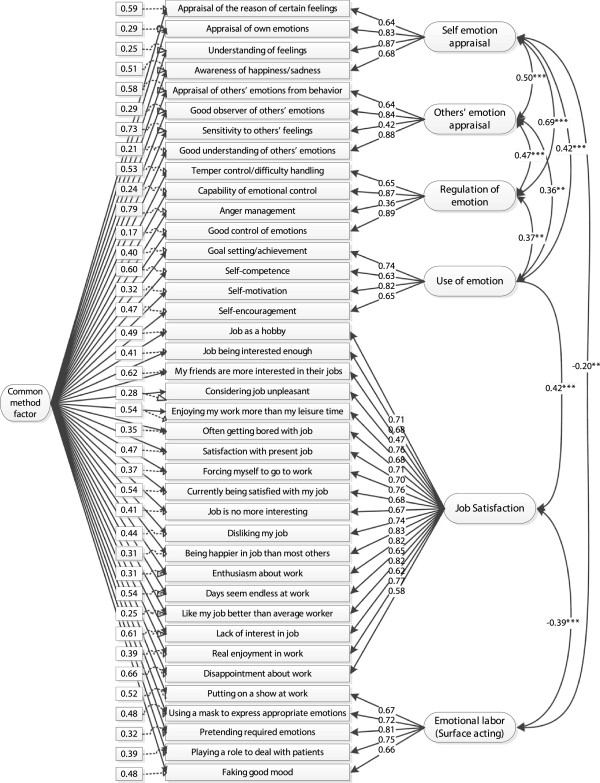
**Standardized solution for the revised model with six correlated factors and one common methods variance factor based on confirmatory factor analysis.** Numbers enclosed in rectangles indicate measurement errors and those in the middle of straight lines indicate factor loadings. Curved lines indicate significant factor correlations (^**^p< .01; ^***^p< .001).

Regarding examination of common method bias, the results of the exploratory factor analysis revealed not a single factor but seven distinct interpretable factors with eigenvalues greater than 1. The seven factors together accounted for 60.91% of the total variance. The first (largest) factor did not account for most of the variance (23.36% in the unrotated solution and 13.65% in the solution after varimax rotation). Thus, no general factor that accounted for most of the covariance among the variables was apparent. Despite the fact that this procedure is widely used to test common method bias, it has several limitations [47]. Consequently, CFA, as a more sophisticated procedure, was employed to test the hypothesis that a single factor could account for most of the variance in our data. All items were allowed to load on their theoretical constructs, as well as on a latent common methods variance factor (Figure 1). Model fit without the latent common methods variance factor was good: root mean square error of approximation (RMSEA)= .04, non-normed fit index (NNFI)= .97, comparative fit index (CFI)= .97, standardized root mean square residual (SRMR)= .08. These fit indices are compatible with those recommended by Hu and Bentler [56] for a good fit to be present between the hypothesized model and the observed data, that is CFI> .95, NNFI> .95, SRMR< .08, and RMSEA< .06. When a latent common methods factor was added to the model, there was a significant improvement in model fit: Δχ^2^(33)= 74.84, *p*< .01 (applying the scaled difference in χ^2^s test for nested models [57]), CFI= .98, NNFI= .98, SRMR= .07, and RMSEA= .03. Thus, the revised model provided a significantly better fit to the data than the original solution without the common methods factor. However, convergent validity and construct reliability of the common methods factor were not supported (CR= .243, AVE= 4.2%), given that the items did not consistently represent the same latent construct and much more error remained in the items than variance explained by the latent factor structure. Only 2.5% of the standardized factor loadings were above .40 for the common methods factor. Furthermore, only 36% of the factor loadings of the manifest variables on the latent common methods factor were significant at 5% level, not satisfying the convergent validity criteria. While the results of these analyses do not preclude the possibility of common method variance, they do suggest that common method variance is not of great concern and thus is unlikely to confound the interpretations of results. 

### Convergent and discriminant validity

Regarding convergent validity for the other factors (i.e., apart from the common methods factor), as shown in Table 1, all constructs exhibited CR values above the conventional threshold of .70, ranging from .800 to .947. AVE criterion (≥ .50) was satisfied for all constructs. Regarding discriminant validity of the constructs, we compared the shared variances between paired factors with the average variance extracted of the individual factors. For example, it can be seen that the AVE of .51 for UOE is greater than the shared variance of .14 (i.e., correlation .37^2^) between UOE and ROE, as well as than the shared variance between UOE and each one of the rest of the constructs. The additional finding that the estimated correlations between the factors were not excessively high (e.g., > .70) further indicated discriminant validity.

**Table 1:** Descriptive statistics, construct reliability, average variance extracted, and intercorrelations for total sample

	**Mean (SD)**	**CR**	**1**	**2**	**3**	**4**	**5**	**6**
1. SEA	22.19 (4.05)	.844	(.58)	.25	.18	.48	.01	.04
2. OEA	21.15 (3.60)	.800	.50^***^	(.52)	.13	.22	.02	.03
3. UOE	20.58 (4.03)	.804	.42^***^	.36^**^	(.51)	.14	.18	.01
4. ROE	19.67 (4.17)	.802	.69^***^	.47^***^	.37^**^	(.53)	.01	.02
5. JS	66.26 (11.32)	.947	.08	.15	.42^***^	.12	(.50)	.15
6. SA	9.75 (4.23)	.846	-.20^**^	-.16	-.08	-.14	-.39^***^	(.52)

Note: *n*=130. Values below the diagonal are correlation estimates among constructs, diagonal elements are construct AVE values (rounded), and values above the diagonal represent squared interconstruct correlations. SD=Standard Deviation, CR=Construct Reliability. ^*^p<.05; ^**^p<.01; ^***^p<.001.

### Correlation analysis

Regarding correlations among the factors, as seen in Table 1, higher doctor EI (UOE dimension) was significantly correlated with more JS (*r*= .42*, p*< .001), while doctors who demonstrated higher levels of SA derived less JS (*r*= −.39, *p*< .001). Hypotheses 1a and 2a were supported. In addition, EI (SEA dimension) was negatively correlated with SA (*r*= −.20*, p*< .01), supporting Hypothesis 3. Correlations among the four dimensions of EI were statistically significant (Table 1).

### Mediation analysis

#### Direct effects

Among the direct effects, significant paths were found from UOE to JS (*B*= −.869, SE= .236, *p*< .001), from SEA to SA (*B*= −.198, SE= .098, *p*= .045), and from SA to JS (*B*= −1.102, SE= .244, *p*< .001). More specifically concerning SEA, the direction of the signs of the path coefficients was consistent with the interpretation that higher SEA led to lower SA (mediator), which in turn led to higher JS.

#### Indirect effects

**Figure 2:**
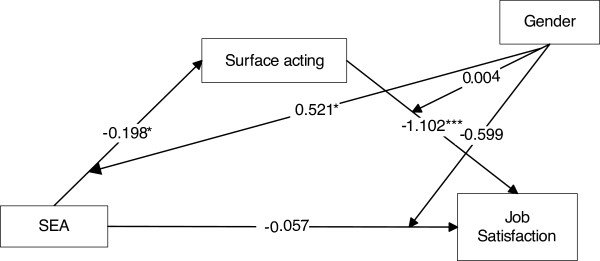
**Mediation model showing the indirect effect of SEA on JS through SA, and moderated mediation model depicting the moderating role of gender.** Values in graph represent unstandardized regression coefficients. (^*^p< .05; ^***^p< .001).

Regarding the indirect effects, SEA was posited to influence JS both directly and indirectly through SA (Figure 2). In the mediation model, SA did mediate the effect of SEA on JS; therefore, hypothesis 4a was supported. The total (direct and indirect) effect was equal to *B*= .157, SE= .228 (*p*= .493), while the indirect effect exerted through SA was equal to *B*= .214, SE= .106 (95% CI= .002, .427). This led to the rejection of the null hypothesis that the indirect effect was zero, given that the corresponding 95% CI did not contain zero. These results did not change substantially when gender and days of duty were included as covariates in the model. No other significant indirect path was found from other EI components (OEA, UOE, ROE) to JS through SA.

### Moderated mediation

We then proceeded to evaluate a moderated mediation model in which the (previously found significant) indirect effect of SEA on JS through SA was presumed to be moderated by gender. The statistically significant interaction between SEA and gender in the model for SA implied that the indirect effect of SEA on JS was moderated by gender (*B*= .521, SE= .258, *p*= .046) (Figure 2). The positive sign of the interaction was consistent with the interpretation that the indirect effect was larger for male physicians (effect= .3386, SE= .151) than female physicians (effect= −.217, SE= .262). Given this significant interaction, it made sense to probe the indirect effect by obtaining bootstrap confidence intervals for these conditional indirect effects. The conditional indirect effect for male physicians was significantly different from 0, at 5% level, given that this interval did not contain 0 (95% CI= .093, .681). This effect was not statistically significant for female physicians (95% CI= −.900, .099). Hypothesis 1b was supported via the indirect SEA-JS path through SA; on the contrary, Hypothesis 2b was not supported, as gender did not moderate the direct effect of SA on JS. No other significant moderated mediation was found for other EI components.

### Moderation analysis

Regarding the moderating role of SA in the relationship between EI and JS, neither the interaction between ROE and SA, nor the interaction of UOE with SA were significant, but the interaction between SEA (and OEA) and SA was. The effects of SEA and OEA on JS were not significant, while their interaction with SA was significant (B= −.120, SE= .045, *p*= .008 and *B*= −.157, SE= .068, *p*= .023, respectively). There was also a significant negative relationship between SA and JS. Using the Johnson-Neyman technique, we found that the moderating effects of SA were significant at 5% level for any value of it greater than 4.401 for SEA, and for values below −1.567 for OEA. Moderation analysis for UOE and ROE revealed no statistically significant results.

**Figure 3:**
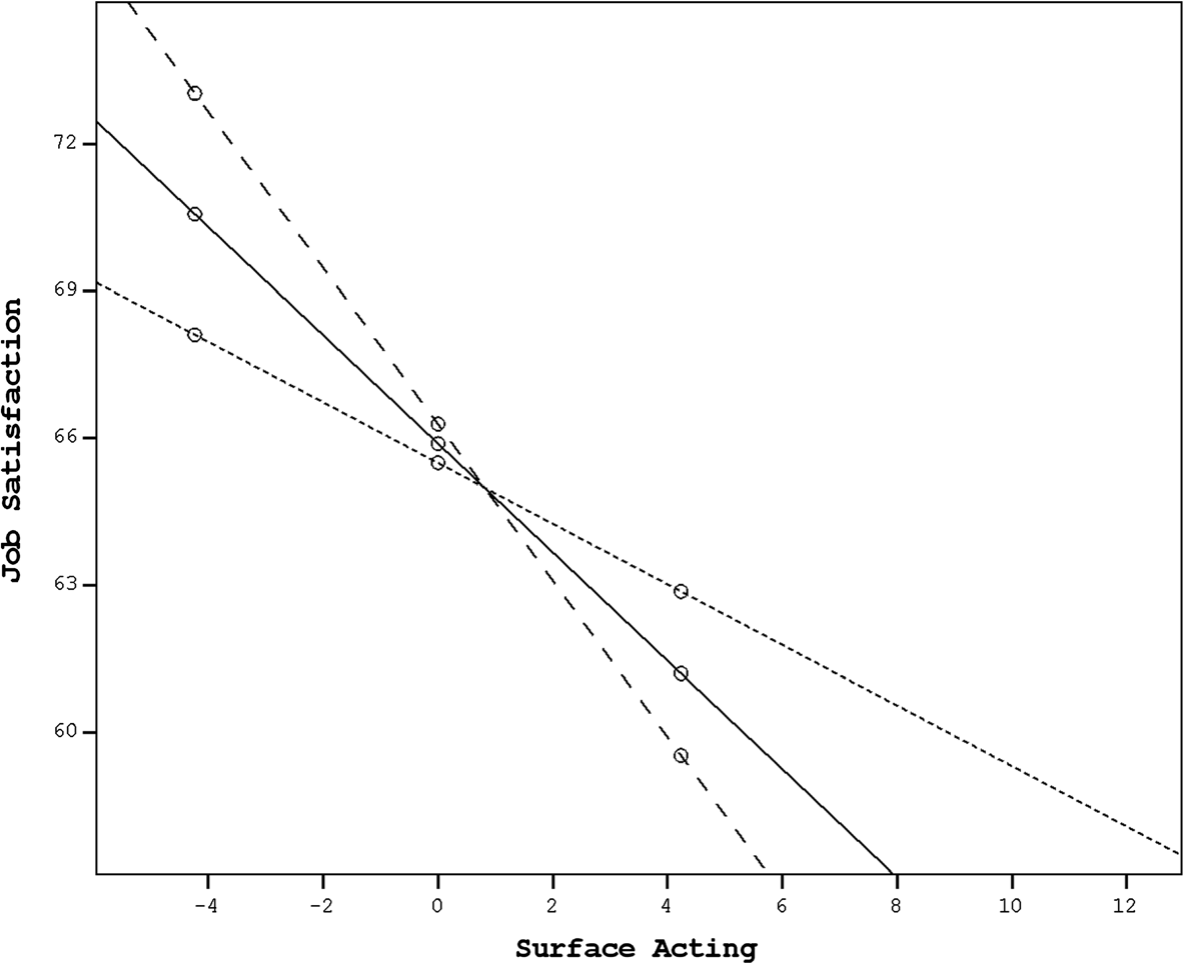
**Moderating role of SA in the relationship between SEA and JS.** The sparsely dotted line corresponds to high SEA levels, the solid line represents moderate SEA levels, and the densely dotted line corresponds to low SEA levels.

The interaction between SEA and SA, depicted visually in Figure 3, was interpreted to mean that, among those physicians with high scores in SA (> 4.401), there was a significant effect of SEA on JS such that physicians with low SEA scores had relatively higher JS compared with those with high SEA scores. Regarding the interaction between OEA and SA, this was interpreted as indicating that among those physicians with low SA scores, there was a significant effect of OEA on JS such that physicians with low OEA scores had relatively low JS compared with those with high OEA scores. Hypothesis 4b was supported.

### Hierarchical multiple regression analysis

Hierarchical- sequential multiple regression analysis was conducted to find out the significant predictors of JS, after controlling for the effects of demographic and work-related variables. Four steps were considered: the demographic variables (e.g., age, gender) were entered first as independent variables, the work-related variables (e.g., days of duty) were added in the second step, the EI variables were entered afterwards, while SA was entered in the fourth step.

Table 2 shows that in the model including SEA as an independent variable, the only variable that appeared to have unstandardized regression coefficients significantly different from zero in predicting JS was SA. The negative coefficient for the SA variable (*B*= −1.138) means that survey physicians who have high SA scores have lower JS, controlling for the other independent variables. The model *R*^2^ when only the demographic variables were in the model was equal to .027. There was a statistically significant improvement (*R*^2^ change= .182) in the relationship between the set of independent variables and the dependent variable, when the SA variable was included. The proportion of variance in the dependent variable explained by the independent variables was 23.2%. The Durbin-Watson statistic was equal to 2.00, a value within the acceptable range of values from 1.50 to 2.50. Multicollinearity was not detected, as none of the independent variables had a small (<.10) tolerance value.

**Table 2:** **Hierarchical multiple regression analysis with JS as the dependent variable, and SEA as one of the predictors (*n*=129)**

**Predictors**	***R***^**2**^	***F*change**	***B***	***SE***	**Beta**	***t***	***p***	**95% CI for*B***	**Tolerance**
Block 1:	.027	F(2,126)=1.748					.178		
*Age*			.414	1.326	.029	.313	.755	−2.210,3.038	.747
*Gender*			−1.945	1.810	-.087	−1.074	.285	−5.529,1.638	.949
Block 2:	.044	F(1,125)=2.188					.142		
*Days of duty*			-.581	.494	-.107	−1.777	.241	−1.558,.396	.753
Block 3:	.050	F(1,124)=.729					.395		
*SEA*			.009	.218	.003	.040	.968	-.422,.440	.944
Block 4:	.232	F(1,123)=29.189					<.001		
*SA*			−1.138	.211	-.443	−5.403	<.001	−1.555,-.721	.930

*Note*. *B*=unstandardized coefficients*; SE*= standard error; Beta (*β*)=standardized coefficients; CI=confidence interval.

Table 3 shows that in the model including UOE as an independent variable, the only variables that appeared to have unstandardized regression coefficients significantly different from zero in predicting JS were SA and UOE. The positive coefficient for the UOE variable (*B*= .894) means that survey physicians who have high UOE scores have high JS as well, controlling for the other independent variables. The model *R*^2^ when only the demographic variables were in the model was equal to .027. There was a statistically significant improvement (*R*^2^ change=.165) in the relationship between the set of independent variables and the dependent variable, when the SA variable was included. The proportion of variance in the dependent variable explained by the independent variables was 33.9%. The Durbin-Watson statistic was equal to 1.84, a value within the acceptable range of values from 1.50 to 2.50. Multicollinearity was not detected, as none of the independent variables had a small (< .10) tolerance value. In addition, it should be noted that SA was the only significant predictor of JS, when the other dimensions of EI, namely OEA and ROE which both had positive regression coefficients in the equation, were introduced as independent variables. Thus, Hypothesis 2c was supported.

**Table 3:** **Hierarchical multiple regression analysis with JS as the dependent variable and UOE as one of the predictors (*n*=129)**

**Predictors**	***R***^**2**^	***F*change**	***B***	***SE***	**Beta**	***t***	***p***	**95% CI for*B***	**Tolerance**
Block 1:	.027	F(2,126)=1.748					.178		
*Age*			.456	1.221	.031	.373	.710	−1.961, 2.873	.758
*Gender*			−2.550	1.674	-.114	−1.524	.130	−5.863, .763	.955
Block 2:	.044	F(1,125)=2.188					.142		
*Days of duty*			-.825	.459	-.152	−1.797	.075	−1.734, .084	.748
Block 3:	.174	F(1,124)=19.633					<.001		
*UOE*			.894	.200	.332	4.472	<.001	.498, 1.290	.972
Block 4:	.339	F(1,123)=30.640					<.001		
*SA*			−1.070	.193	-.416	−5.535	<.001	−1.453, -.687	.950

*Note*. *B* =unstandardized coefficients*; SE*=standard error; Beta (*β*)=standardized coefficients; CI= confidence interval.

## Discussion

The results of this study indicate that physicians with higher EI (i.e., higher UOE) have higher levels of JS. This finding is in accordance with a growing number of studies, showing a positive correlation between EI and JS [13,14,19,41]. EI may promote the building of interpersonal relationships in the work environment [13] and contribute to an employee’s success and competence in an organization [20]. Furthermore, awareness of the factors that elicit particular emotions, positive or negative, permits employees to act in the most appropriate way to enhance job satisfaction [14]. Physicians receiving collegial support and maintaining long-term relationships with patients are more satisfied [58]. Therefore, physicians who use the practical skills underlying EI (i.e. self-confidence, empathy, adaptability, conflict management) [15] to successfully interact with patients and coworkers may feel more competent and satisfied with their job.

Additionally, a negative correlation between SA of EL and JS was observed, i.e. the more physicians displayed an appropriate but not felt emotion in their interpersonal relationships with colleagues and patients, the less satisfied with their job they were. It has been supported that EL can undermine JS by increasing emotional demands, thereby contributing to increased levels of stress and psychological distress as well as symptoms of depression of the employee [30-32]. The present study focused on the SA component of EL, as there is increasing evidence that the negative effects of EL, such as stress and job dissatisfaction, are mediated by SA (as opposed to deep acting) [29,35,38].

Our findings confirm the mediating role of SA in the relation between SEA dimension of EI and JS. In the present study, the supported model of partial mediation, i.e. only a part of the total effect of EI on JS is due to SA, is not surprising as EI is also considered to have a direct effect on JS. On the other hand, other variables such as positive and negative affect [13], and self-esteem [59] have been reported to function as mediators in the EI-JS relationship.

SA was also found to moderate EI-JS relation. With increased SA, physicians with low SEA scores had relatively higher JS compared with those with high SEA scores. As far as OEA dimension of EI is concerned, with decreased SA scores, physicians with low OEA scores had relatively low JS compared with those with high OEA scores. Hochschild [30] supported that the extent of EL may differ across occupations. Based on that, Wong and Law [44] hypothesized, although did not prove, that the EI-JS relationship was moderated by the extent of EL; EI is expected to have greater effect on JS for employees who engage more frequently and extensively in the process of EL. EI constitutes a key asset to performing effectively a job requiring high levels of discrepancy between expressed and experienced emotions (e.g. social worker, nurse, physician); yet its significance may be less important for jobs involving little EL.

Although moderation and mediation are distinct processes, a variable may function both as a moderator and a mediator in a single functional relation [60]. However, to the best of our knowledge, a possible moderating and/or mediating role of SA has not been sufficiently tested in the EI-JS relation in medicine. In addition to both the moderating and mediating roles, SA was found to be a predictor of JS variable, above and beyond EI dimensions. This finding can be explained in the context of the high EL inherent in clinical practice [61] and point out SA as a source of strain undermining physicians’ professional satisfaction and well-being.

Gender was not found to moderate the direct effect of SA on JS. However, the indirect effect of SEA on JS, via SA, was moderated by gender, with this positive effect being larger for male physicians than their female counterparts. According to previous studies, females are more likely to experience job dissatisfaction when engaged in SA [39]; on the other hand, the effect of EI on JS may be fully mediated by positive and negative affect for men but partially for women [13]. Gender’s moderating role may be interpreted in the context of gender differences in the hospital workplace, such as responsibilities, family- and work-related stressors experienced by female physicians, who simultaneously take on the roles of mothers and professionals, and gender-specific resistance to females’ effort to ascend organizational hierarchies [22].

Research has revealed a negative relationship between EI and SA [40,42]. In the present study, physicians with high SEA were less likely to mask their true emotions in order to comply with organizational display rules. This finding could be related to the superior abilities attributed to high-EI individuals, such as understanding their true emotions and expressing emotions naturally [7,44,62].

The use of self-report measures, which may result in response bias (e.g. social desirability, mood state) and in overstatement of the relationships between the examined constructs [47], does serve as limitations of the study. Furthermore, the adopted cross-sectional research design of our study renders difficult any interference about the causative nature of the examined relationships. Additionally, the power to detect moderators might have been decreased by the relatively small sample size, unequal sample sizes across groups (e.g. male vs. female), and possibly heterogeneous error variance [51]. Other limitations of our study concern the collection of data from one hospital center and the relative brevity of the measures used.

Elucidating interactions between emotion-related constructs and job satisfaction is critical to developing support programs and communication-skill training courses that may facilitate emotional appraisal and emotional regulation, reduce the related individual and organizational costs, and contribute to the improvement of health care quality. In addition, personality constructs (i.e. EI) might be used as predictive variables for health care managers in order to recruit physicians who would be most effective in the emotionally “charged” hospital environment [63]. Emotion management workshops and interpersonal skill training could be incorporated in medical schools’ curriculum with a view to preparing more competent doctors.

Despite the extensive literature on EI and JS, scant research has integrated EI with SA and JS, particularly in the hospital workplace. Most empirical studies, in health care environment, have examined the role of emotion-related constructs in the “nursing framework” [64], although a strong emotional component is interwoven with medical profession, as well [65]. Doctors interact with people at one of the most important or difficult circumstances of their lifetime and are often required to take on complex, albeit not always harmonized with their true experienced emotions, roles. This study provides evidence on the interactions between emotion-related constructs, presenting an integrative EI-SA-JS model. Further research based on longitudinal design, larger sample sizes across different health care settings and encompassing methods based on physiology (e.g. monitoring heart rate during the performance of emotional labor) is needed to examine in more depth the influence of emotions in the workplace, the causal associations, and the effect of emotion-related parameters on physicians’ wellbeing, on delivered patient care and on organizational management.

## Conclusions

In conclusion, emotionally intelligent physicians seem to be more satisfied with their job and this positive relation is moderated and partially mediated by SA component of EL. Additionally, the more physicians display an appropriate but not felt emotion in their interpersonal relationships with colleagues and patients, the less satisfied with their job they are. The findings of the present study could help to clarify aspects of the emotional dimensions of health care with a view to improving the quality of service in the health sector.

## Abbreviation

EI: Emotional Intelligence; EL: Emotional Labor; SA: Surface Acting; JS: Job Satisfaction; OEA: Others’ Emotion Appraisal; ROE: Regulation of Emotion; SEA: Self-Emotion Appraisal dimension; UOE: Use of Emotion; WLEIS: Wong Law Emotional Intelligence Scale; CFA: Confirmatory Factor Analysis; AVE: Average Variance Extracted; CR: Construct Reliability; SD: Standard Deviation; SE: Standard error; CI: Confidence interval.

## Competing interests

The authors declare that they have no competing interests.

## Authors’ contributions

The present study was carried out by all authors working collaboratively. AP conceived the study, collected data, performed statistical analysis, and wrote the first draft of the paper. FA performed advanced statistical analyses, participated in data interpretation, and revised the paper. EM contributed to the design and analysis of the data. DN participated in data interpretation and was involved in revising the manuscript critically. All authors have contributed to, seen and approved the manuscript.

## Pre-publication history

The pre-publication history for this paper can be accessed here:

http://www.biomedcentral.com/1472-6963/12/463/prepub
